# Health‐related quality of life in patients with metastatic basal cell carcinoma treated with cemiplimab: Analysis of a phase 2 trial

**DOI:** 10.1002/cam4.7360

**Published:** 2024-07-19

**Authors:** Ketty Peris, Timothy J. Inocencio, Alexander J. Stratigos, Karl D. Lewis, Zeynep Eroglu, Anne Lynn S. Chang, Cristina Ivanescu, Aleksandar Sekulic, Matthew G. Fury, Chieh‐I Chen, Ruben G. W. Quek

**Affiliations:** ^1^ Catholic University Fondazione Policlinico Universitario‐IRCCS Rome Italy; ^2^ Regeneron Pharmaceuticals, Inc. Tarrytown New York USA; ^3^ National and Kapodistrian University of Athens, Andreas Sygros Hospital Athens Greece; ^4^ Department of Cutaneous Oncology Moffitt Cancer Center Tampa Florida USA; ^5^ Dermatology Department Stanford University School of Medicine Redwood City California USA; ^6^ IQVIA Durham North Carolina USA; ^7^ Mayo Clinic Scottsdale Arizona USA

**Keywords:** cemiplimab, EORTC QLQ‐30, functioning, health‐related quality of life, metastatic basal cell carcinoma, Skindex‐16

## Abstract

**Background:**

A phase 2 cemiplimab study (NCT03132636) demonstrated a 24.1% objective response rate in patients diagnosed with metastatic basal cell carcinoma (mBCC) who were not candidates for continued hedgehog inhibitor (HHI) therapy due to intolerance to previous HHI therapy, disease progression while receiving HHI therapy, or having not better than stable disease on HHI therapy after 9 months. Here, health‐related quality of life (QoL) for this patient population is reported.

**Methods:**

Adult patients with mBCC were treated with intravenous cemiplimab at a dose of 350 mg every 3 weeks for 5 treatment cycles of 9 weeks/cycle then 4 treatment cycles of 12 weeks/cycle. Patients completed the European Organisation for Research and Treatment of Cancer Quality of Life‐Core 30 (QLQ‐C30) and Skindex‐16 questionnaires at baseline and Day 1 of each cycle. Across Cycles 2 to 9, the overall change from baseline was analyzed using a mixed model with repeated measures. Responder analyses determined clinically meaningful improvement or deterioration (changes ≥10 points) or maintenance across all scales.

**Results:**

Patients reported low symptom burden and moderate‐to‐high functioning at baseline. Maintenance for QLQ‐C30 global health status (GHS)/QoL and across all functioning and symptom scales was indicated by overall mean changes from baseline. Clinically meaningful improvement or maintenance was reported at Cycle 2 for GHS/QoL (77%), functioning scales (77% to 86%), and symptom scales (70% to 93%), with similar proportions of improvement or maintenance at Cycles 6 and 9, excluding fatigue. On the Skindex‐16, clinically meaningful improvement or maintenance was reported across the emotional, symptom, and functional subscales, in 76%–88% of patients at Cycle 2, which were generally maintained at Cycles 6 and 9. Overall mean changes from baseline showed maintenance across these subscales.

**Conclusions:**

The majority of patients treated with cemiplimab reported improvement or maintenance in GHS/QoL and functioning while maintaining a low symptom burden.

## 1 INTRODUCTION

Basal cell carcinoma (BCC) remains the most common type of cancer, as well as the most common type of skin cancer, but its prevalence is likely underestimated, as BCC is generally excluded from reporting and tracking in cancer registries.^1^, ^2^ Surgical intervention is the cornerstone of treatment in the majority of cases, due to the high risk of recurrence and the necessity for histopathological analysis.^3^, ^4^ Radiotherapy has been reported as an alternative in cases where surgery is not appropriate.^5^ Early diagnosis and surgery are associated with a favorable prognosis in most patients with common BCC; however, a small proportion of cases may progress to unresectable locally advanced BCC (laBCC) or metastatic BCC (mBCC). While classified as a rare disease, mBCC (with an estimated incidence of 0.028%–0.55%) is associated with a poor prognosis and is considered difficult to treat as therapeutic options are limited.^6^, ^7^, ^8^, ^9^ Treatment guidelines recommend the use of hedgehog signaling pathway inhibitors (HHIs) in patients with mBCC, especially those for whom surgery and radiotherapy are not appropriate or could be potentially disfiguring.^7^, ^8^ Two HHIs that have received regulatory approval in the US and EU as treatment of laBCC and mBCC are vismodegib and sonidegib.^4^, ^7^ However, their utility is limited by side effects such as muscle spasms, dysgeusia, and alopecia; furthermore, resistance to HHIs is common,^6^, ^10^ suggesting the need for alternative therapies in these patients.

Cemiplimab (known as cemiplimab‐rwlc in the US) is approved in the US and EU for the treatment of patients with laBCC and mBCC who have progressed on HHI treatment or for whom HHIs are not appropriate.^11^, ^12^ In a pivotal phase 2 trial, cemiplimab demonstrated safety and efficacy in patients with laBCC and mBCC.^13^ Treatment with cemiplimab in patients with mBCC resulted in an overall response rate of 24.1% (95% confidence interval [CI] 13.5–37.6%), based on Response Evaluation Criteria in Solid Tumors version 1.1 (complete response + partial response).^14^

Investigational drugs for mBCC are in development that aim to overcome the limitations of currently approved interventions, for example, topical HHI formulations (notably patidegib)^15^ or HHIs that target components of the hedgehog signaling pathway other than those targeted by vismodegib and sonidegib (i.e., oral itraconazole, taladegib, patidegib, and silmitasertib).^3^ However, trials that have explored the potential of these new therapies have not yet resulted in approvals for the treatment of BCC.^3^, ^15^

The use of patient‐centered outcomes, especially for evaluating the effects of therapy on health‐related quality of life (HRQoL) from the patient's perspective, has become increasingly recognized by healthcare stakeholders including managed care and regulatory authorities. In particular, the US Food and Drug Administration and the European Medicines Agency have noted the importance of such outcomes and have provided guidance on their use in clinical trials.^16^, ^17^ Additionally, a position statement by the European Academy of Dermatology and Venereology recommends the use of both cancer‐ and dermatology‐specific patient‐reported outcomes (PROs) for the assessment of HRQoL.^18^ Accordingly, assessment of HRQoL was included in the cemiplimab trial and showed that cemiplimab resulted in maintenance of HRQoL and a low symptom burden over the duration of treatment in patients with laBCC.^19^ The current analysis reports the results of HRQoL assessment in patients with mBCC.

## 2 MATERIALS AND METHODS

### 2.1 Study design and population

This open‐label, non‐randomized, multicenter, phase 2 clinical trial evaluated the treatment of cemiplimab monotherapy in adult patients (≥18 years old) with mBCC or laBCC (clinicaltrials.gov identifier NCT03132636; EudraCT number 2016‐003122‐16). The study was conducted in accordance with the Declaration of Helsinki and each participating study site received approval by the institutional review board; written informed consent was provided by all patients.

The mBCC cohort consisted of patients with histologic confirmation of distant BCC metastases to lung, liver, bone, or lymph node who were not candidates for continued HHI therapy due to intolerance to previous HHI therapy, disease progression while receiving HHI therapy, or having not better than stable disease on HHI therapy after 9 months. The cohort included patients with both nodal and distant metastatic disease, and patients were also required to have an Eastern Cooperative Oncology Group (ECOG) performance status of 1 or 0.

### 2.2 Treatment

Treatment was cemiplimab 350 mg, administered intravenously every 3 weeks for 5 treatment cycles of 9 weeks/cycle, followed by 4 treatment cycles of 12 weeks/cycle. Patients were treated until the 93‐week treatment period was completed or until unacceptable toxicity, disease progression, or withdrawal of consent, whichever occurred first.

### 2.3 Patient‐reported outcomes

Patient‐reported HRQoL, which was a predefined secondary endpoint, was measured using the European Organisation for Research and Treatment of Cancer (EORTC) Quality of Life Questionnaire Core 30 (QLQ‐C30)^20^ and the Skindex‐16^21^ questionnaires at baseline and Day 1 of each treatment cycle. Follow‐up assessment with these PROs was conducted 28–42 days after the last study treatment administration if a patient discontinued early.

The QLQ‐C30 is a 30‐item general oncology PRO tool that assesses HRQoL with a recall period of “during the past week.” It consists of a global health status (GHS)/quality of life (QoL) scale, functional domain scales, and symptom scales. The GHS/QoL is assessed on a 7‐point Likert scale, and functional domains and symptoms are assessed on a 4‐point Likert scale. All scores are subsequently transformed to a 0–100 scale; better outcomes are denoted by high scores on GHS/QoL and functional scales and low scores on the symptom scales. The Skindex‐16 is a 16‐question dermatology‐specific PRO tool that assesses the effect of skin disease on patients' HRQoL based on symptom (4 items), emotional (7 items), and functional (5 items) subscales. The 16 items measure the level of bother over the previous week on a 7‐point Likert scale (0 = never bothered to 6 = always bothered). Scores are transformed to a scale from 0 to 100, where lower scores indicate improved outcomes.

### 2.4 Statistical analysis

Analyses were conducted on the full analysis set, defined as all enrolled patients with mBCC who were deemed eligible for the study. Completion rates for the QLQ‐C30 and Skindex‐16 were calculated for those patients who were expected to complete the instrument (those who were alive and receiving study treatment). A mixed model for repeated measures (MMRM) analysis estimated least‐squares (LS) mean change from baseline with a 95% CI on all scales of the QLQ‐C30 and Skindex‐16 for patients with both a baseline and at least 1 post‐baseline value. The MMRM analyses used all data available with the assumption that missing observations were missing at random. Scores of domains with missing items were calculated according to the EORTC scoring algorithm (i.e., ≥50% of the items were required for the domain score calculation).^21^ The overall change from baseline on each scale was evaluated across Cycles 2–9. Covariates that were included in the model were time, baseline score, a timepoint*baseline score interaction term, age group, sex, ECOG performance status, number of prior systemic therapies, and geographic region. Two‐sided nominal *P‐*values were calculated with no adjustments for multiple comparisons; significance was set at *α* = 0.05.

Among patients with non‐missing data, a responder analysis was conducted to determine the proportions who reported an improvement or deterioration from baseline that was clinically meaningful, or maintenance of HRQoL at Cycles 2, 6, and 9; Cycle 6 represents 54 weeks (approximately 1 year) of treatment. Based on published literature, a difference in absolute value was considered clinically meaningful for both the QLQ‐C30^22^, ^23^, ^24^ and the Skindex‐16,^25^, ^26^ if the change from baseline was 10 or more points. Maintenance was defined as a change from baseline that was not clinically meaningful. Analyses were conducted using SAS version 9.4 (SAS Institute, Cary, NC, USA).

## 3 RESULTS

### 3.1 Patient population

Of the 54 patients with mBCC who met the entry criteria and were enrolled, 70.4% were male, and the mean age ± standard deviation (min, max) was 63.8 ± 11.1 (38, 90) years (Table 1). Two‐thirds of these patients (66.7%) had an ECOG performance status of 0, and disease progression was the primary reason cited for discontinuation of prior HHI therapy (75.9%).

**TABLE 1:** Patient characteristics at baseline (*n* = 54).

Variable	Value
Age, mean ± SD, (min, max) years	63.8 ± 11.1 (38, 90)
≥65, *n* (%)	27 (50.0)
Male, *n* (%)	38 (70.4)
Race, *n* (%)	
White	47 (87.0)
Not reported	1 (1.9)
Missing	6 (11.1)
BMI, mean ± SD, kg/m^2^	26.2 ± 5.6
ECOG performance status score, *n* (%)	
0	36 (66.7)
1	18 (33.3)
Time from initial diagnosis to first study treatment dose, mean ± SD, months	117.5 ± 110.5
Stage at first known diagnosis, *n* (%)	
I	2 (3.7)
II	3 (5,6)
III	4 (7.4)
IV	11 (20.4)
Unknown	30 (55.6)
Primary tumor site, *n* (%)	
Trunk	25 (46.3)
Head and neck	22 (40.7)
Extremity	6 (11.1)
Anogenital	1 (1.9)
Metastatic status, *n* (%)	
Distant and nodal	29 (53.7)
Distant only	19 (35.2)
Nodal only	5 (9.3)
Reason for discontinuation of prior HHI, *n* (%)^a^	
Disease progression	41 (75.9)
Intolerance	18 (33.3)

Abbreviations: BMI, body mass index; ECOG, Eastern Cooperative Oncology Group; SD, standard deviation.

^a^ Sum is >54 because some patients had more than 1 reason for discontinuation.

Baseline completion rates for the QLQ‐C30 and Skindex‐16 in the total mBCC cohort were >90% for all scales. For every treatment cycle through Cycle 7, >81% and >76% of patients completed at least 1 question on the QLQ‐C30 and Skindex‐16, respectively.

### 3.2 QLQ‐C30

**FIGURE 1:**
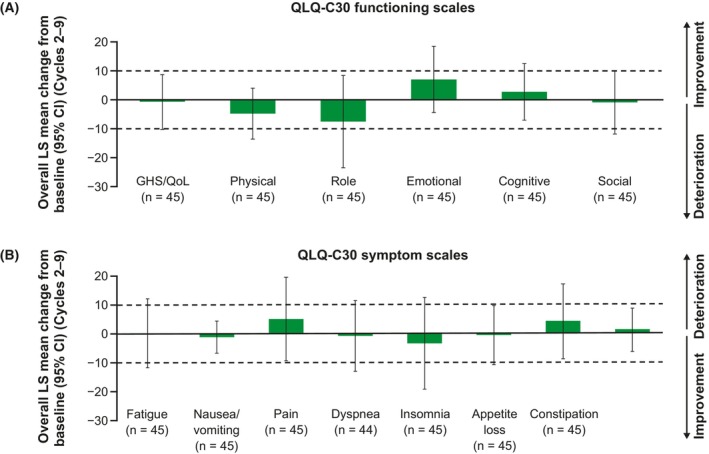
Overall change from baseline (MMRM) on (A) the QLQ‐C30 functioning scales and (B) the symptom scales in patients in the full analysis set who had both a baseline and at least 1 post‐baseline value. Dashed horizontal lines indicate the threshold for a clinically meaningful change. CI, confidence interval; GHS, global health status; LS, least squares; MMRM, mixed model with repeated measures; QLQ‐C30, Quality of Life Questionnaire Core 30; QoL, quality of life.

As shown in Table 2, patients reported low symptom burden and moderate‐to‐high levels of functioning at baseline as indicated by QLQ‐C30 scores, which were slightly better than reference values for overall cancer patients.^27^ In the MMRM analysis, the overall changes from baseline across the study period indicated maintenance on the GHS/QoL scales (LS mean change −0.7, 95% CI –10.2 to 8.9) and all functioning scales (Figure 1A); these changes were neither clinically meaningful (<10‐point change) nor statistically significant (“0” was included within the 95% CI). Similarly, the MMRM analysis showed all symptom scale scores were maintained across the study period, including fatigue which had the smallest change from baseline among the symptom scales (LS mean change 0.1, 95% CI –11.6 to 11.9) (Figure 1B).

**TABLE 2:** Baseline scores on the QLQ‐C30 among patients in the full analysis set who had both a baseline and at least 1 post‐baseline assessment, relative to reference values for general cancer patients.^27^

QLQ‐C30 scale	Mean (SD)	
	Study population (*n* = 45)	Reference^a^
GHS/QoL	67.0 (22.5)	61.3 (24.2)
Functional scale		
Physical	83.8 (20.4)	76.7 (23.2)
Role	79.3 (28.0)	70.5 (32.8)
Emotional	76.9 (27.1)	71.4 (24.2)
Cognitive	86.3 (20.2)	82.6 (21.9)
Social	81.5 (23.9)	75.0 (29.1)
Symptom scale		
Fatigue	27.2 (25.4)	34.6 (27.8)
Nausea/vomiting	4.1 (10.2)	9.1 (19.0)
Pain	30.7 (32.2)	27.0 (29.9)
Dyspnea	9.1 (16.6)^b^	21.0 (28.4)
Insomnia	28.9 (31.5)	28.9 (31.9)
Appetite loss	11.1 (21.3)	21.1 (31.3)
Constipation	8.9 (16.5)	17.5 (28.4)
Diarrhea	3.7 (10.6)	9.0 (20.3)

*Note*: Better outcomes are indicated by high scores on the GHS/QoL and functional scales, and low scores on the symptom scales.

Abbreviations: GHS, global health status; QLQ‐C30, Quality of Life‐Core 30; QoL, quality of life; SD, standard deviation.

^a^ Reference values from EORTC all cancer patients: all stages.^27^

^b^ *n* = 44.

**FIGURE 2:**
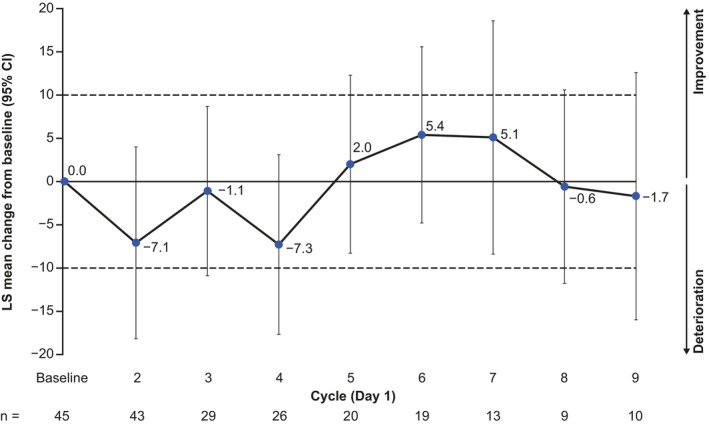
MMRM analysis of change from baseline for the QLQ‐C30 GHS/QoL scale by treatment cycle in patients in the full analysis set who had both a baseline and at least 1 post‐baseline value. Dashed horizontal lines indicate the threshold for a clinically meaningful change. CI, confidence interval; GHS, global health status; LS, least squares; MMRM, mixed model with repeated measures; QLQ‐C30, Quality of Life‐Core 30; QoL, quality of life.

At each time point, the change from baseline in GHS/QoL scores also suggested that overall HRQoL was generally maintained across the study duration, with no changes that were clinically meaningful or statistically significant (Figure 2).

**FIGURE 3:**
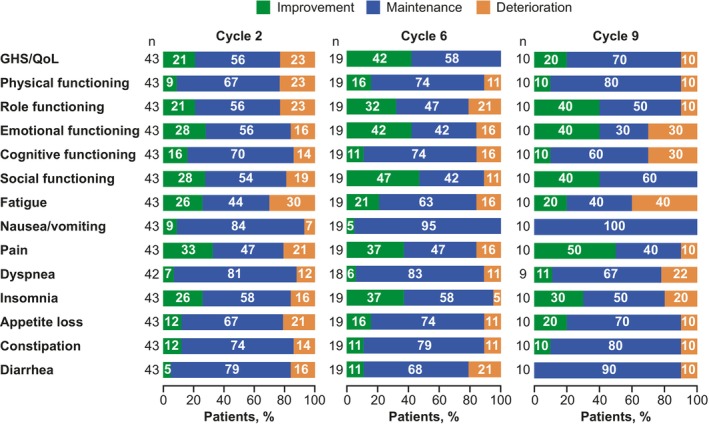
Proportion of patients reporting clinically meaningful improvement, maintenance, or deterioration on the QLQ‐C30 at Cycles 2, 6, and 9. GHS, global health status; QLQ‐C30, Quality of Life‐Core 30; QoL, quality of life.

Clinically meaningful improvement or maintenance on all scales in the responder analysis, was reported by most patients at Cycle 2, including 77% of patients on the GHS/QoL, with ranges of 77%–86% and 70%–93% of patients on the functioning and symptom scales, respectively (Figure 3). Similar proportions were reported after approximately 1 year of treatment (Cycle 6); results were consistent at Cycle 9 except for fatigue, with 60% of patients reporting clinically meaningful improvement or maintenance.

### 3.3 Skindex‐16

**FIGURE 4:**
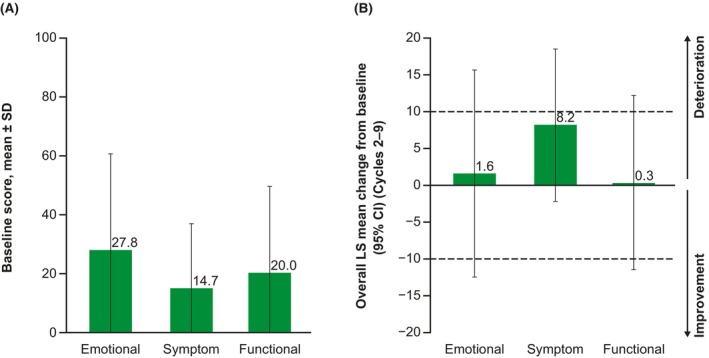
(A) Baseline scores^a^ and (B) MMRM overall change from baseline on the Skindex‐16 among patients in the full analysis set who had both a baseline and at least 1 post‐baseline assessment (*n* = 43). ^a^Lower scores indicate better outcomes. Dashed horizontal lines indicate the threshold for a clinically meaningful change. CI, confidence interval; LS, least squares; MMRM, mixed model with repeated measures; SD, standard deviation.

**FIGURE 5:**
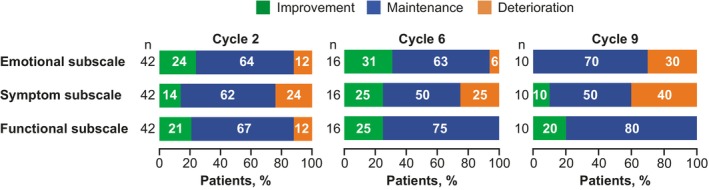
Proportion of patients reporting clinically meaningful improvement, maintenance, or clinically meaningful deterioration on the Skindex‐16 at cycles 2, 6, and 9.

The Skindex‐16 baseline scores for the emotional, symptom, and functional subscales are shown in Figure 4A, and the overall LS mean changes from baseline across Cycles 2–9 are shown in Figure 4B. While the largest overall change was on the symptom subscale, all three subscales were characterized by maintenance, with no clinically meaningful or statistically significant changes relative to baseline. Clinically meaningful improvement or maintenance at Cycle 2 was reported by 76%–88% of patients across all 3 subscales (Figure 5) in the responder analysis. Similar proportions were generally observed at Cycles 6 and 9.

## 4 DISCUSSION

While survival outcomes for patients with mBCC have improved due to new therapeutic modalities,^6^, ^9^ it is also important to determine whether clinical benefits in this difficult‐to‐treat population are accompanied by outcomes of importance to patients, such as function and HRQoL.^28^ Assessing PROs is supported by the European Society for Medical Oncology Magnitude of Clinical Benefit Scale guidance as part of a more comprehensive approach to evaluating the value of treatment in clinical trials, in addition to efficacy and safety.^29^ This study is the first to evaluate PROs in patients with mBCC treated with cemiplimab. The results reported here provide evidence that the objective outcomes of clinically meaningful antitumor activity in patients with mBCC treated with cemiplimab^14^ are complemented by maintenance of HRQoL and moderate‐to‐high levels of functioning; no significant differences in overall changes from baseline on the QLQ‐C30 GHS/QoL, function scales, and the 3 Skindex‐16 scales were observed. These patient‐reported benefits, assessed in accordance with recommendations for the use of both cancer‐ and dermatology‐specific measures,^18^ were obtained while also preserving a low symptom burden. The responder analysis further supports these benefits on all outcomes: the majority of patients reported maintenance or clinically meaningful improvement with generally similar proportions at Cycles 2, 6, and 9, representing almost 2 years of treatment.

The high level of functioning at baseline in this population was comparable to that in the laBCC population,^19^ likely as a result of the inclusion criterion of ECOG performance status 0 or 1. While baseline GHS/QoL and functioning scores on the EORTC QLQ‐C30 and symptom burden on the Skindex‐16 were similar to those reported for patients with laBCC,^19^ patients with mBCC showed a slightly higher symptomatology on the EORTC QLQ‐C30 than those with laBCC, reflecting greater disease severity of mBCC relative to laBCC. It is therefore relevant to note that, despite the higher symptom burden, there was no additional clinically meaningful deterioration in any of the symptoms. Additionally, the EORTC QLQ‐C30 indicated that patients with mBCC experienced a greater amount of pain at baseline than those with laBCC, which may have impacted QoL in this study.

Maintenance of a low symptom burden is consistent with the safety and tolerability profile that has been previously reported for cemiplimab in patients with laBCC and mBCC^1^, ^13^, ^14^; immune‐related treatment‐emergent adverse events of Grade ≥3 occurred in 5 (9%) patients with mBCC.^14^ In addition, as previously reported, fatigue was the most frequent adverse event reported (per common terminology criteria for adverse events) with cemiplimab,^14^ and was essentially unchanged from baseline in the MMRM analysis (per patient‐reported EORTC QLQ‐C30). However, in the responder analysis, the highest proportion of patients with clinically meaningful deterioration at Cycle 2 and Cycle 9 (30% and 40%, respectively) was linked to the symptom of fatigue (per patient‐reported EORTC QLQ‐C30), thus suggesting that a subpopulation of patients may be more affected by fatigue as has also been observed for those with laBCC.^19^

Results on the Skindex‐16 were concordant with those observed on the QLQ‐C30; none of the 3 Skindex‐16 subscales was characterized by a clinically meaningful or statistically significant overall change from baseline. While the greatest disease impact at baseline was on the emotional subscale, there was little change in the overall analysis; most patients (≥70%) reported clinically meaningful improvement or maintenance at Cycles 2, 6, and 9. The functional subscale also showed minimal overall change, further emphasizing maintenance of function in this patient population.

These results are consistent with those observed in patients with mBCC in clinical trials of HHIs,^26^, ^30^ suggesting that treatment with cemiplimab provides patient‐reported benefits in HRQoL, functioning, and symptom burden despite previous progression or intolerance to HHIs.

Limitations of this analysis include the single‐arm, non‐randomized, open‐label study design and the small sample size, especially in the final treatment cycles (≤10 patients), that may limit data interpretability at these time points. While the open‐label design has the potential to introduce response bias, as outcomes in this analysis were PROs there is a lack of clear empirical evidence that such bias is sufficient to meaningfully affect the results of clinical trials.^31^ Even though there was a high rate of completion of the study PROs, the results over‐represent patients who do well since those who progress may not necessarily complete the questionnaires. While the fact that anchor‐based approaches to derive clinically meaningful changes within the trial population were not performed may also be construed as a limitation, our definition of clinically meaningful changes was based on published literature.^22^, ^23^, ^24^, ^25^, ^26^ Similarly, to the best of our knowledge and in contrast to the QLQ‐C30, reference values have not been determined for the Skindex‐16. An additional limitation is that, mainly as a result of the small sample size, patient‐reported results were not stratified by clinical response or tumor location; previous studies of HHIs suggest that both of these variables likely affect HRQoL.^26^, ^32^ Finally, the missing‐at‐random assumption used in the MMRM analysis advocates the similarity of withdrawals and those who remain in the study. However, this may not be realistic, as discontinuation is often due to disease progression.

## 5 CONCLUSION

Results of this analysis in patients with mBCC show that treatment with cemiplimab, in addition to providing clinically meaningful anti‐tumor activity and durable response,^14^ provides stability in patient‐reported HRQoL. From baseline to Cycle 9, most patients who received cemiplimab treatment maintained a low symptom burden while reporting maintenance or improvement in QLQ‐C30 GHS/QoL and functioning. The benefits of treatment on HRQoL‐related outcomes were additionally demonstrated by maintenance of scores across the emotional, symptom, and functional subscales on the Skindex‐16. When combined with previous observations in patients with laBCC, the results of this pivotal clinical trial expand the evidence in support of cemiplimab for patients with advanced BCC who progress on or are intolerant to HHIs, or for whom HHI therapy may not be appropriate. Further studies, including those that capture PROs in real‐world clinical settings, would help confirm the HRQoL results reported here.

## AUTHOR CONTRIBUTIONS

**Ketty Peris:** Formal analysis (equal); investigation (equal); writing – review and editing (equal). **Timothy J. Inocencio:** Conceptualization (equal); formal analysis (equal); methodology (equal); writing – review and editing (equal). **Alexander J. Stratigos:** Formal analysis (equal); investigation (equal); writing – review and editing (equal). **Karl D. Lewis:** Formal analysis (equal); investigation (equal); writing – review and editing (equal). **Zeynep Eroglu:** Formal analysis (equal); investigation (equal); writing – review and editing (equal). **Anne Lynn S. Chang:** Formal analysis (equal); investigation (equal); writing – review and editing (equal). **Cristina Ivanescu:** Conceptualization (equal); formal analysis (equal); methodology (equal); writing – review and editing (equal). **Aleksandar Sekulic:** Formal analysis (equal); investigation (equal); writing – review and editing (equal). **Matthew G. Fury:** Conceptualization (equal); formal analysis (equal); writing – review and editing (equal). **Chieh‐I Chen:** Conceptualization (equal); formal analysis (equal); methodology (equal); writing – review and editing (equal). **Ruben G. W. Quek:** Conceptualization (equal); formal analysis (equal); methodology (equal); writing – review and editing (equal).

## FUNDING INFORMATION

This study was sponsored by Regeneron Pharmaceuticals, Inc., and Sanofi.

## CONFLICT OF INTEREST STATEMENT

Ketty Peris reports advisory board roles with AbbVie, Almirall, Galderma, Janssen, Leo Pharma, Lilly, Novartis, Pierre Fabre, Sanofi, and Sun Pharma. Timothy J. Inocencio, Karl D. Lewis, Matthew G. Fury, Chieh‐I Chen, and Ruben G.W. Quek report employment as well as stock and other ownership interests at Regeneron Pharmaceuticals, Inc. Alexander J. Stratigos reports advisory board or steering committee roles with Janssen Cilag, Regeneron Pharmaceuticals, Inc., Roche, and Sanofi; and research support from AbbVie, Bristol‐Myers Squibb, Genesis Pharma, and Novartis. Zeynep Eroglu reports participation in advisory boards for Array, Eisai, Genentech, Natera, Novartis, OncoSec, Pfizer Canada Inc., and Regeneron Pharmaceuticals, Inc.; and research funding from Boehringer Ingelheim, Pfizer, and Novartis. Anne Lynn S. Chang reports being an advisory board member for Sun Pharma, Regeneron Pharmaceuticals, Inc., Merck, and Novartis; a consultant for Feldan and Castle; and a clinical investigator for Regeneron Pharmaceuticals, Inc., Merck, and Novartis. Cristina Ivanescu reports employment at IQVIA; and stock and other ownership interests at IQVIA. Aleksandar Sekulic reports advisory roles with Regeneron Pharmaceuticals, Inc., and Roche.

## ETHICS STATEMENT

This study was done in accordance with the principles of the Declaration of Helsinki and with Good Clinical Practice guidelines as defined by the International Conference on Harmonization.

## PATIENT CONSENT STATEMENT

All patients provided written, informed consent before enrolment.

## Data Availability

Qualified researchers may request access to study documents (including the clinical study report, study protocol with any amendments, blank case report form, and statistical analysis plan) that support the methods and findings reported in this manuscript. Individual anonymized participant data will be considered for sharing once the product and indication has been approved by major health authorities (eg, US Food and Drug Administration, European Medicines Agency, Pharmaceuticals and Medical Devices Agency, etc.), if there is legal authority to share the data and there is not a reasonable likelihood of participant re‐identification. Requests should be submitted to https://vivli.org/.
